# A retrospective review of patients with significant traumatic brain injury transported by emergency medical services within the south east of England

**DOI:** 10.29045/14784726.2019.03.3.4.1

**Published:** 2019-03-01

**Authors:** Jack William Barrett

**Affiliations:** South East Coast Ambulance Service NHS Foundation Trust

**Keywords:** ambulance, older, trauma network, traumatic brain injury, triage

## Abstract

**Introduction:**

Traumatic brain injury (TBI) will be a leading cause of death and disability within the Western world by 2020. Currently, 80% of all TBI patients in England are transported to hospital by an ambulance service. The aim of this retrospective study is to compare TBI patients transported to a major trauma centre (MTC) against those transported to a trauma unit (TU).

**Method:**

All patients with a primary injury of TBI who were transported to hospital by South East Coast Ambulance Service NHS Foundation Trust (SECAmb) from 1 January 2016 to 31 December 2016 and entered into the Trauma Audit & Research Network (TARN) registry were reviewed. Patients were stratified by hospital designation (MTC or TU). Severity of TBI was categorised using the patients’ pre-hospital Glasgow Coma Scale (GCS) and Abbreviated Injury Score (AIS) Head. The outcomes of interest were 30-day mortality and Glasgow Outcome Score (GOS) at discharge.

**Results:**

Between 1 January and 31 December 2016, 549 TBI patients were identified in the TARN database as being transported by SECAmb to either an MTC or a TU. The majority of patients were transported to a TU (77.96%), and the median age of the TU cohort was older than the MTC group (TU 82.15 IQR 16.73 vs. MTC 62.1 IQR 42.6). The median Injury Severity Score (ISS) was greater in the MTC cohort (22 IQR 10 vs. 17 IQR 9), where falls from height and road traffic collisions (RTCs) contributed to 50.51% of all injuries. Within the TU cohort, falls from less than 2 metres (standing height) were the main mechanism of injury (MOI) (77.62%). The median length of hospital stay (LOS) was longer in the MTC cohort compared to the TU cohort (10 IQR 13.25 vs. 8 IQR 14).

**Conclusion:**

The high proportion of mild TBI and absence of reliable triage guidelines make it difficult for ambulance clinicians to identify patients who will benefit from transport to an MTC. Future research should focus on how TBI triage influences outcomes and how ambulance services can better identify patients with a TBI and who would benefit from specialist care.

## Introduction

In the UK there are approximately 1.2 million cases of traumatic brain injury (TBI) seen in the emergency department (ED) every year (NICE, 2014). Emergency medical services (EMS) are responsible for transporting 80% of TBI patients from the scene of the injury to an ED (Lawrence et al., 2016), which means that EMS have a significant influence on which hospitals TBI patients are transported to.

In Western societies, the incidence of TBI and its socioeconomic burden has become significant, and by 2020 the World Health Organization predicts that it will be one of the leading causes of death and disability (Hydar, Wunderlich, Puvanachandra, Gururaj, & Kobusingye, 2007). Historically, TBI has been an injury of the young due to road traffic collisions (RTC), violent assaults and sporting injuries. However, despite the continued rise of TBI, the incidence of RTCs, assaults and sporting injuries has remained constant (Roozenbeek, Maas, & Menon, 2013; Salottolo et al., 2017; Walder et al., 2013). This apparent disparity has been attributed to the increasing incidence of TBI in older adults.

The recognition of significant trauma in older adults (defined in this article as patients ≥ 65 years) over the last 20 years has contributed to the growing TBI problem (Kehoe, Smith, Edwards, Yates, & Lecky, 2015b). The body region most commonly injured is the head (> 70% of all major trauma), while falls of less than 2 metres are the most common cause of major trauma in England and Wales (Trauma Audit & Research Network, 2017a). These injuries, in part, are due to older patients’ co-morbidities, anticoagulant medications and the natural physical decline seen with age (Walder et al., 2013).

Following a restructure of trauma care in the UK, ambulance services now have access to a network of regional level one major trauma centres (MTC) with neurosurgical facilities, and local trauma units (TUs), to manage patients with traumatic injuries (National Audit Office, 2010; Sleat & Willett, 2011). However, current trauma decision tools lack the sensitivity to accurately aid pre-hospital triage of TBI patients (Fuller, Lawrence, Woodford, & Lecky, 2014) and recent attempts to test new criteria have resulted in the over-triage of patients with TBI (Lecky et al., 2017).

While research has been able to explore the pre-hospital management and triage of severe TBI (defined as a head injury with a Glasgow Coma Scale [GCS] ≤ 8) (Davis et al., 2003; Ochs et al., 2002), there is a paucity of research in the pre-hospital triage and management of mild (GCS 13–15) and moderate (GCS 9–12) TBI. This is an important area to explore further, especially in the older TBI patient who is likely to present with a high level of consciousness despite having a significant TBI.

The aim of this study was to describe patients with significant TBI presenting to a regional UK ambulance service, which hospital these patients were transported to (MTC or TU) and whether a difference in outcomes could be observed.

## Methods

South East Coast Ambulance Service NHS Foundation Trust (SECAmb) covers a geographical area of approximately 3000 square miles, with a population of approximately four million across the English counties of Kent, Sussex, Surrey and North East Hampshire. The region has a mix of densely populated urban towns, rural villages, motorway networks and air- and sea-ports. This presents an operational challenge to ensure members of the public get a timely response from their EMS provider. SECAmb are supported by a network of MTCs with neurosurgical specialities (n = 4) and TUs (n = 15) across the Trust. SECAmb operates a 24/7 service and employs around 2000 front line staff; paramedics and advanced paramedics make up 53% of this workforce. The remaining 47% of clinical support staff consists of ambulance technicians and emergency care support workers, responding from 14 operational units across the Trust. Critical care support can also be provided by Kent, Surrey and Sussex Helicopter Emergency Medical Services.

A research request was submitted to the Trauma Audit & Research Network (TARN) seeking use of retrospective data between 1 January and 31 December 2016. The TARN registry includes patients admitted to hospital for 72 hours or more, admitted to a critical care unit, who die within a hospital or who are transferred to another hospital (Trauma Audit & Research Network, 2017b).

Patients eligible for inclusion were those who had been transported to hospital by SECAmb and met the following criteria: a trauma patient with a TBI as their primary injury, whose CT head scan revealed significant TBI (Abbreviated Injury Score [AIS] Head ≥ 3). Patient data included pre-hospital GCS, mechanism of injury (MOI), Injury Severity Score (ISS), co-morbidities, Glasgow Outcome Score (GOS) and length of hospital stay (LOS). Patients who received a secondary transfer were also included in the dataset.

TBI severity was categorised using the patient’s pre-hospital GCS against the TBI severity scale (mild TBI GCS 15–13, moderate TBI GCS 12–9 and severe TBI ≤ 8) (Kehoe, Rennie, & Smith, 2015a). Patients were grouped according to the hospital they were transported to (MTC or TU), and comparisons were made between the two cohorts. The primary outcomes of interest were 30-day mortality and GOS at discharge.

## Results

Between 1 January and 31 December 2016, 549 TBI patients were identified in the TARN database as being transported by SECAmb to either an MTC or TU. The majority of patients were transported to a TU (77.96%), and the median age of the TU cohort was older than for the MTC group (TU 82.15 IQR 16.73 vs. MTC 62.1 IQR 42.6). The median ISS was greater in the MTC cohort (22 IQR 10 vs. 17 IQR 9), where falls from height and RTCs contributed to 50.51% of all injuries. Within the TU cohort, falls from less than 2 metres (standing height) were the main MOI (77.62%). The median LOS was longer in the MTC cohort compared to the TU cohort (10 IQR 13.25 vs. 8 IQR 14). Table 1 summarises the patient demographics and characteristics between the two cohorts.

**Table 1.** Comparison between the major trauma centre and trauma unit cohorts.

	MTC		TU	
n (%)	121	(22.04)	428	(77.96)
Median age (IQR)	62.60	(42.60)	82.15	(19.19)
< 18 years n (%)	6	(4.96)	9	(2.10)
18–65 n (%)	63	(52.07)	74	(17.29)
65 > n (%)	52	(42.98)	345	(80.61)
Males n (%)	82	(67.77)	222	(51.87)
Median ISS n (IQR)	22	(18.18)	17	(3.97)
TBI				
Mild n (%)	86	(71.07)	372	(86.92)
Moderate n (%)	17	(14.05)	33	(7.71)
Severe n (%)	17	(14.05)	18	(4.21)
Unknown n (%)	1	(0.83)	5	(1.17)
AIS Head				
3 n (%)	33	(27.27)	71	(16.59)
4 n (%)	54	(44.63)	215	(50.23)
5 n (%)	34	(28.10)	142	(33.18)
6 n (%)	0	(0.00)	0	(0.00)
MOI				
Blast n (%)	1	(0.83)	0	(0.00)
Blow n (%)	13	(10.74)	19	(4.44)
Crush n (%)	1	(0.83)	0	(0.00)
Falls less than 2 m n (%)	45	(37.19)	331	(77.34)
Falls more than 2 m n (%)	25	(20.66)	5	(11.68)
Other n (%)	0	(0.00)	8	(1.87)
RTC n (%)	36	(29.75)	20	(4.67)
Co-morbidities	67	(55.37)	380	(88.79)
Atrial fibrillation n (%)	2	(1.65)	92	(21.50)
Asthma n (%)	4	(3.31)	21	(4.91)
Cancer n (%)	9	(7.44)	52	(12.15)
CKD n (%)	0	(0.00)	40	(9.35)
COPD n (%)	4	(3.31)	36	(8.41)
Stroke/TIA n (%)	3	(2.48)	40	(9.35)
Hypertension n (%)	22	(18.18)	174	(40.65)
IHD n (%)	0	(0.00)	40	(9.35)
T1DM n (%)	6	(4.96)	4	(0.93)
T2DM n (%)	7	(5.79)	58	(13.55)
Length of stay n = days (IQR)	10	(8.26)	8	(1.87)
30-day mortality n (%)	14	(11.57)	71	(16.59)

Figures in brackets are a percentage of the total number in their respective cohort. AIS: abbreviated injury score; CKD: chronic kidney disease; COPD: chronic obstructive pulmonary disease; IHD: ischemic heart disease; ISS: injury severity score; MOI: mechanism of injury; RTC: road traffic collision; T1DM: type 1 diabetes mellitus; T2DM: type 2 diabetes mellitus; TIA: transient ischemic attack.

Mild TBI was the most common TBI presentation in both cohorts, however a greater proportion of mild TBI patients were transported to a TU (MTC 71.01% vs. TU 86.4%) (Table 1). Conversely, the proportion of moderate and severe TBI was greater in the MTC cohort compared to the TU cohort (moderate TBI: MTC 14.05% vs. TU 7.71%; severe TBI: MTC 15.05% vs. TU 4.21% patients). Furthermore, the AIS scoring system was used to measure TBI severity: AIS 4 (severe) was the most common finding in both the MTC and TU cohorts (43.9% and 50.47%).

Computer tomography (CT) scans reported on seven different types of TBI (Table 2), with a proportion of patients sustaining more than one type of injury. Patients in the MTC group presented with a higher proportion of multiple TBIs compared to the TU group (56.20% vs. 31.54%). There was a higher proportion of subarachnoid haemorrhage (SAH) in the TU cohort compared to the MTC group (33.64% vs. 24.73%, respectively). Equally, subdural haematoma (SDH) was a more common presentation in the TU cohort compared to the MTC cohort (60.75% vs. 45.2%). Skull fractures were found in over half of the MTC cohort (54.8%) compared to the TU group (23.1%).

**Table 2.** Comparison of type of traumatic brain injury found on computer tomography head scans.

Head CT scan findings	MTC (n = 121)		TU (n = 428)	
Single TBI n (%)	53	(43.80)	293	(68.46)
Multiple TBI n (%)	68	(56.20)	135	(31.54)
Extradural haematoma n (%)	4	(3.30)	31	(7.24)
SDH n (%)	28	(23.1)	260	(60.75)
SAH n (%)	30	(24.74)	144	(33.64)
Diffuse axonal injury n (%)	2	(1.65)	1	(0.23)
Contusion n (%)	48	(39.67)	83	(19.39)
Simple skull fracture n (%)	48	(39.67)	71	(16.59)
Complex skull fracture n (%)	22	(18.18)	22	(5.14)

Figures in brackets are a percentage of the total number in their respective cohort. CT: computer tomography; MTC: major trauma centre; SAH: subarachnoid haemorrhage; SDH: subdural haematoma; TBI: traumatic brain injury; TU: trauma unit.

In total, six (1.09%) patients received a neurosurgical intervention and were all taken directly to an MTC. There were 23 (5.37%) secondary transfers from a TU to an MTC, however none of these patients received a neurosurgical intervention. Based on the GOS (Table 3), the proportion of patients making a good recovery was almost equal between the cohorts (MTC 37.19% vs. TU 37.85%). The GOS findings were also reflected in the patient discharge destination described in Table 4.

**Table 3.** A comparison of Glasgow Outcome Score between the two cohorts.

Glasgow Outcome Score	MTC		TU	
Good recovery n (%)	45	(37.19)	163	(38.08)
Moderate disability n (%)	4	(3.31)	52	(12.14)
Severe disability n (%)	0	–	7	(1.63)
Persistent vegetative state n (%)	0	–	0	–
Death n (%)	14	(11.57)	76	(17.75)
Not available n (%)	58	(47.93)	130	(30.37)
Total n (%)	121	(100)	428	(100)

Figures in brackets are a percentage of the total number in their respective cohort. MTC: major trauma centre; TU: trauma unit.

**Table 4.** Discharge destinations of patients between the two cohorts.

Discharge destination	MTC		TU	
Home (own) n (%)	44	(36.36)	158	(36.92)
Home (carer or relative) n (%)	0	–	6	(1.4)
Mortuary n (%)	14	(11.57)	72	(16.82)
Nursing home n (%)	1	(0.83)	38	(8.88)
Other acute hospital n (%)	1	(0.83)	9	(2.1)
Other institution n (%)	0	–	1	(0.23)
Rehabilitation n (%)	2	(1.65)	11	(2.57)
Unavailable n (%)	59	(48.76)	133	(31.07)
Total n (%)	121	(100)	428	(100)

Figures in brackets are a percentage of the total number in their respective cohort. MTC: major trauma centre; TU: trauma unit.

## Discussion

The results of this retrospective review of TBI patients conveyed to hospital by SECAmb suggest that the majority of significant TBI (AIS Head ≥ 3) patients with a high functioning GCS are transported to a TU. Patients taken to an MTC were younger, subjected to injuries with higher forces and had fewer co-morbidities. Conversely, patients in the TU cohort were predominantly older, victims of falls from standing height and had a greater number of recorded pre-existing co-morbidities. In both cohorts TBI was predominantly mild (MTC 66.1% vs. TU 86.4%), however when TBI severity was graded using AIS Head, 80.06% (*n* = 445/539) of all patients had an AIS score of four or more. In the TU cohort 83.41% (*n* = 357/428) had a severe or critical AIS score. However, only 1.09% of patients (*n* = 6/539) received a neurosurgical intervention, and these patients were all transported to an MTC.

A small number of patients were transported from a TU to an MTC (*n* = 23/539, 5.37%); none of these secondary transfers received a neurosurgical intervention, however these patients may have benefitted from a secondary transfer for direct specialist care (Patel, Woodford, King, Yates, & Lecky, 2005).

Accurate triage guidelines for head injured patients are not currently available to pre-hospital clinicians. The head injury transportation straight to neurosurgery (HITS-NS) cluster randomised controlled trial used head injury specific criteria (isolated head injury, reduced GCS of 14 or lower, no airway compromise) to transport patients to a local ED or to by-pass to one with neurosurgical facilities (Lecky et al., 2017). Based on these criteria, Lecky and colleagues demonstrated an over-triage of TBI by 4:1 and neurosurgically important TBI by 13:1. Despite this, 30-day outcomes were similar between the intervention and control arms. Interestingly, paramedics in the intervention arm were only compliant in 69% of recruited patients compared to 90% in the control arm. Paramedics over-estimated the distance to the by-pass hospital and believed that the patient’s injury was not severe enough to warrant by-pass.

Older patients represented 80.61% of the TU cohort compared to 42.98% in the MTC cohort. This study does not have sufficient data to explain why older patients were more likely to be conveyed to a TU or whether they were appropriately triaged. While TARN does indicate whether a patient is triage positive/negative, it is based on the decision tools mentioned earlier. This is problematic for two reasons. Firstly, in the UK most ambulance services use the trauma decision tree developed by the London Ambulance Service. Both this and the HITS-NS decision tree have shown low sensitivity for patients with a significant TBI head (AIS > 3) (44.5% [95% CI 43.2 to 45.9] and 32.6% [95% CI 31.4 to 33.9], respectively) (Fuller et al., 2014). An alternative is the NICE head injury guidelines, however these advise whether a patient should be referred to EMS or the ED and, if appropriate, a head CT scan (NICE, 2014). NICE do not provide guidance to ambulance services on whether a patient should go to an acute hospital with neurosurgical facilities. A lack of guidance in the management and triage of TBI patients could explain why a high proportion are being transported to TUs. Secondly, evidence from Newgard et al. (2011), who explored triage in older trauma patients, found biases that influenced paramedic decision-making when assessing older trauma patients. Paramedics were more likely to use their intuition rather than follow a trauma algorithm and experienced paramedics tended to forgo the use of a decision tree altogether, believing it was only for newly qualified staff. This was compounded by negative feedback from receiving hospitals when older patients were over-triaged by EMS staff.

In older TBI patients, the severity of their injury is not correlated to the likelihood of death or disability. Salottolo et al. (2017) noted that in younger adults, mortality was linked to abnormal vital signs, compared to normal vital signs (51% vs. 27%, p < 0.001). In contrast, older patients presenting with normal vital signs had a similar mortality to those with abnormal vital signs (48% vs. 46%, p = 0.92). Walder et al. (2013) reported on similar findings, and found that the pre-hospital GCS was likely to be lower for younger patients compared to their older counterparts for the same severity of TBI. Walder and colleagues suggest that one reason for this difference is the MOI. Young adults are more often victims of high kinetic force, causing significant injuries to develop quickly. In contrast, older adults experience lower kinetic forces and intracranial injuries develop at a lower rate. The differences between old and young TBI patients have been widely supported in the literature (Adelic et al., 2012; Andriessen et al., 2011; Bayen et al., 2013; Ellis, Davies, Pearn, & Lockey, 2007; Kehoe et al., 2015a, 2016; Patel et al., 2005).

Kehoe et al. (2015a) have highlighted how GCS is unreliable when measuring TBI severity. While the majority of TU patients in this study would be classed as having a mild TBI based on their GCS, their TBI was classed as serious to critical according to AIS Head. The patients’ GCS reflects their behavioural change to injury compared with the AIS that grades the severity of the TBI based on CT findings (Scheetz, Horst, & Arbour, 2018).

Without the availability of reliable point-of-care head scanning (POCHS), pre-hospital clinicians are unable to define AIS Head (Brogan, Kontojannis, Garara, Marcus, & Wilson, 2017). Microwave-based technology is currently being explored as a means to detect intra-cranial haemorrhage in the acute head injury, measuring the dialectic properties of a patient head (Persson et al., 2014). In a phantom trial, Candefjord et al. (2017) demonstrated that a head scanning device using microwaves was able to detect subdural hematomas of various sizes (0, 40, 70 and 110 ml), with a reported sensitivity and specificity of 100% and 96%, respectively. If the technology was accurate enough to measure the size of the bleed and its location, a more accurate TBI diagnosis may be given by pre-hospital clinicians, which could influence the delivery of care and the triage TBI patients receive.

A small proportion of TBI patients in this study received neurosurgery (*n* = 6/549, 1.09%), however it is unclear why these patients were eligible candidates. These findings could be a reflection of the reservation about treating TBI patients with surgery, especially older patients because of their poor prognosis (De Bonis et al., 2011), while others have cited a high (13–15) or low GCS as a reason not to surgically intervene (Herou, Romner, & Tomasevic, 2015). Conversely, other authors have argued that a conservative approach contributes to a poor prognosis in TBI patients and that aggressive management, in the older patients, can improve outcomes, although more research is required in this area (Wan et al., 2016).

There was a small number of TU patients (*n* = 23/428, 5.37%) who received a secondary transfer to an MTC despite none of these patients receiving a neurosurgical intervention. It has been observed that mild TBI patients with an SAH do not need to be managed directly by neurosurgical teams (Levy, Orlando, Salottolo, Mains, & Bar-Or, 2014). However, other authors have argued that patients may benefit from direct care from neurosurgical teams without surgical intervention (Harrison et al., 2013). Neurosurgical units are a limited and costly resource. Identifying who would benefit from these resources could allow for better management and prognosis of TBI patients. Whether this decision is the responsibility of EMS providers or the trauma network is subject to debate and further research.

The proportion of patients who made a good recovery, measured using GOS, was similar between the two cohorts (Table 3: MTC 37.19% vs. TU 38.08%); this was also reflected in the discharge destinations (Table 4), with an equal proportion returning to their home (MTC 36.36% vs. TU 36.92%). Patients transported to an MTC were likely to be younger, with few co-morbidities and subjected to MOIs with potentially higher kinetic forces. This is a stark contrast to the patients transported to TUs who are older, with pre-existing medical conditions and subjected to lower kinetic forces.

These findings highlight that in the south-east region of England, 77.96% of patients with significant TBI are transported to TUs. An audit performed by the London Major Trauma System found that only 33% of patients with a significant TBI were cared for at an MTC (London Major Trauma System, 2018). While TUs will discuss patients with neurosurgeons at their partner MTC within the network, the TU is principally responsible for the care delivered to the patient. The findings from this study suggest that TUs are capable of providing the appropriate care TBI patients require based on proportionally similar outcomes, however this requires significant exploration.

### Limitations

This was a retrospective review of a registry database regarding one ambulance service in the UK. TARN is reliant on accurate recording and entry of patient observations, treatments and outcomes. These data were missing in some cases and this could have influenced the results. These findings may not be generalisable to all UK ambulance services, however the prevalence of older adults suffering low velocity falls is consistent with the wider literature. Poor patient outcomes in this study were due to all causes and not mutually exclusive to TBI. A substantial amount of missing GOS data limits meaningful comparison between the MTC and TU cohorts; furthermore, the lack of data meant that multi-variable analysis was not possible. Although mortality was notably worse in the TU cohort, this group of patients was older, with more co-morbidities, limiting meaningful comparison. The clinical reason for neurosurgical intervention and secondary transfer was not available, therefore it is difficult to understand the rationale for these events. Finally, it is unclear which of the patients who attended MTCs were true by-pass patients and who attended because it was the closest hospital. However, it should be acknowledged that of the four MTCs available to SECAmb, only one is in its geographical region, in an urban area of the south coast. Two are located in south London and another in Hampshire. While some patients may have attended an MTC because it was their closest ED, it is likely many had to by-pass their local ED.

## Conclusion

A high proportion of TBI patients are transported to hospital via the ambulance service (Lawrence et al., 2016), placing the responsibility for ensuring the right patient is conveyed to the right hospital in the hands of EMS personnel. Currently, this is challenging to achieve as the patient’s GCS does not necessarily correspond to the underlying severity of the TBI as scored by AIS Head (Kehoe et al., 2015a, 2016). The high proportion of mild TBI and absence of reliable triage guidelines make it difficult for ambulance clinicians to triage those patients who will benefit from transport to an MTC. Future research should focus on how TBI triage influences outcomes and how ambulance services can better identify patients with a TBI and who would benefit from specialist care.

## Acknowledgements

Two anonymous peer reviewers made helpful suggestions in the presentation of this article. The author would like to thank Professor Fiona Lecky, Professor David Yates, Mike Young and Gemma Reed of the Trauma Audit & Research Network for the support in acquiring and using the data for this article. Finally, thanks to Professor Julia Williams (SECAmb Research Lead) for her helpful comments and suggestions in the development of the manuscript.

## Conflict of interest

The author has no financial, personal or honorary affiliations with any commercial organisation directly involved with or discussed in this study.

## Ethics

Separate ethics committee approval for this study was not required as TARN already has ethical approval in place for the use of its data for research purposes. Permissions were sought from the South East Coast Ambulance Service NHS Foundation Trust Research and Development Department to undertake this review and publish the findings.

## Funding

None.
